# Investigation of structural, electronic, optical, and photocatalytic properties of new double perovskites Cs_2_InSbX_6_ (X = F, Cl) under strain effects

**DOI:** 10.1016/j.heliyon.2024.e40315

**Published:** 2024-11-12

**Authors:** S. Tariq, L.H. Omari, F. Mezzat, E.K. Hlil

**Affiliations:** a LPMAT, Faculty of Sciences Ain-Chock, Hassan II University of Casablanca, Morocco; b LaMCScI, URL-CNRST, Faculty of Sciences, University Mohammed V of Rabat, Morocco; c Grenoble Alpes University, CNRS, Grenoble INP, Néel Institute, 38000, Grenoble, France

**Keywords:** Cs_2_InSbX_6_, DFT calculation, Stain engineering, Effective masses, Optoelectronic properties, Photocatalytic properties

## Abstract

Inorganic halide perovskites, such as Cs₂InSbX₆ (X = F, Cl), have shown potential for next-generation solar cells and photocatalysis due to their light-capturing properties. However, concerns about stability and toxicity in lead-based perovskites drive the need for eco-friendly alternatives like antimony-based compounds. This study employed Density Functional Theory (DFT) calculations to explore how mechanical strain affects the electronic and optical properties of Cs₂InSbX₆. Without strain, Cs₂InSbF₆ and Cs₂InSbCl₆ exhibit bandgaps of 2.06 eV and 1.20 eV, respectively. We investigated how strain ranging from −5% to +5 % influences these materials' performance in optoelectronic and photocatalytic applications. Notably, negative strain enhances absorption and quantum efficiency for both compounds, particularly Cs₂InSbCl₆, which shows superior absorption in the visible spectrum. Our findings suggest that Cs₂InSbCl₆, with a tunable bandgap and favorable optical characteristics under strain, is a promising candidate for environmentally friendly applications, including solar cells and photocatalysis.

## 1 Introduction

The global demand for sustainable energy solutions has fueled a surge in research on perovskite materials. These promising materials hold immense potential as light absorbers in solar cells, paving the way for a more ecofriendly future. Beyond photovoltaics, perovskites versatility extends to various high-performance electronic and optical devices. Rapid advancements in this field are driven by the exploration of novel material compositions, innovative structural designs, and cutting-edge manufacturing methods. This progress holds significant promise for addressing the world ever-growing energy demands [[1], [2], [3], [4]]. Over the past years, halide perovskite solar cells have garnered significant attention due to their remarkable power conversion efficiency, surpassing 25 % [5]. Nonetheless, several significant challenges still persist in utilizing perovskites for solar cell and optoelectronic detector fabrication [[6], [7], [8], [9]], primarily attributed to the presence of lead, a toxic element. Lead adverse environmental impact and its compound susceptibility to degradation under moisture and temperature further intensify the issue. Addressing lead toxicity requires substituting Pb^2+^ with elements that are less toxic, non-toxic, or environmentally benign. In this context, various alternatives have been proposed. Firstly, the substitution of Pb^2+^ with elements that share similar electronic configurations, such as Sn^2+^ and Ge^2+^, has been suggested [10]. Secondly, the application of double perovskites with a general formula Cs_2_B^+^B^+3^X_6_, where B^+^ and B^+3^ predominantly represent transition metals, is being explored to replace 2 Pb^2+^ ions. For instance, the work of McClure et al. [11] highlights that synthesized Cs_2_AgBiCl_6_ and Cs_2_AgBiBr_6_ compounds display indirect band-gap energies of 2.77 eV and 2.19 eV, respectively. These materials have been endorsed as potential candidates for photovoltaic applications due to their robust stability in ambient air [12]. Furthermore, as reported by Meng et al., double perovskites based on B^+^ (In, Tl) and B^3+^ (Bi, Sb) have emerged as promising absorbers for solar cells, attributed to their extended carrier recombination lifetimes, impressive stability against air and humidity, and notable direct band-gap energies [13]. The primary challenges involve identifying stable halide perovskites with a narrow bandgap for efficient absorption within the visible spectrum. Notably, a recent advance has been achieved by substituting Bismuth with Indium in Cs_2_AgBiCl_6_, leading to the prediction and synthesis of Cs_2_InAgCl_6_, a novel stable halide double perovskite (H-DP) featuring a direct bandgap of up to 3.3 eV [14]. Additionally, this compound has demonstrated photosensitivity, undergoing a reversible transformation from white to orange upon exposure to ultraviolet light. On the other hand, as previously indicated, the Cs_2_AgBiX_6_ (X = Cl or Br) perovskite demonstrates stability, effective light absorption, and a prolonged carrier recombination lifetime [15,16]. However, its indirect band structure and slightly larger band gap could potentially constrain its application in photovoltaics [17]. Trivalent ions, such as Sb^3+^, In^3+^, and Tl^3+^, can substitute the Bi^3+^ ion to yield various types of double perovskites. For instance, Cs_2_AgSbCl_6_ and Cs_2_AgInCl_6_ exhibit indirect and direct band gaps, respectively. Moreover, alloying the two types of trivalent ions can facilitate a transition between indirect and direct band gaps [18,19].

Similarly, this concept can be extended to encompass monovalent ions. Alkali metal ions such as Cu^+^, Au^+^, and In^+^. Also, Tl^+^ can be substituted with Ag^+^. The materials Cs_2_BB'Cl_6_, which incorporate both trivalent and monovalent ions from the main group elements, are also viable options. Extensive computational studies have delved into the possibilities offered by trivalent and monovalent ions from the main group elements. As a result of these investigations, several double perovskites, including Cs_2_InBiBr_6_, Cs_2_TlAsI_6_, and Cs_2_TlSbBr_6_, have emerged as potential candidates. These selections are underpinned by their maximum limited spectroscopic efficiency (SLME) at 1 μm [20]. Interestingly, two direct band gap materials, Cs_2_InB'Cl_6_ (where B′ can be Sb or Bi), exhibit band gap values of around 1.0 eV and demonstrate efficiencies comparable to those achieved by MAPbI_3_ [21,22]. Furthermore, an investigation has been carried out into the optoelectronic and thermoelectric properties of the halide double perovskites Cs_2_InSbX_6_ (X = Cl, Br, I), revealing their notable attributes and potential suitability for various commercial energy applications [23]. Lead-based perovskites have gained considerable attention due to their excellent optoelectronic properties. However, their widespread use is hindered by concerns over lead toxicity, posing significant environmental and health risks [24]. To address this issue, antimony (Sb) has emerged as a promising alternative to lead (Pb), offering comparable optoelectronic and thermoelectric properties without the associated toxicity concerns. In particular, compounds like Cs₃Sb₂I₉ and CsInSbAgX₆ (X = Cl, Br, and I) not only demonstrate both chemical and mechanical stability but also exhibit a certain level of ductility, making them highly suitable for various applications. This substitution retains the desirable characteristics of lead-based materials while eliminating toxicity concerns, thus offering a more sustainable and safer alternative for next-generation devices [[24], [25], [26]]. The unique optical and electrical properties of double perovskites can be attributed to several factors, including their low effective mass, strong excitonic binding energy, direct bandgap character, and advantageous optoelectronic features, as revealed by this theoretical analysis. Investigating the thermodynamic attributes of these materials is crucial, especially for perovskites, as it allows to predict their behavior under various conditions. Insights into the thermodynamic stability of double perovskites provide valuable information on their response to variations in temperature, pressure, or environmental conditions. This understanding can help prevent unfavorable phase transitions or performance deterioration. It enables optimal use and longevity of these materials in various applications. Moreover, examining the thermodynamic properties enables the identification of optimal compositions that can achieve tailored bandgaps matching the solar spectrum, ultimately enhancing energy conversion efficiencies. In this study, by employing DFT calculations, electronic properties of Cs_2_InSbX_6_ (X = F, Cl) under mechanical stress were investigated. The strain range (−5% to +5 %) is considered because it is appropriate to induce a modification in electronic structure, that induces the physical properties change, without any change in crystallographic structure (space group).

Primary focus was on exploiting the X-ion-mediated bandgap tuning for assessing their potential in optoelectronics and thermodynamics. Computational results revealed notable feature where the bandgap of these materials undergoes a transformation from a broader to a narrower range upon introducing X ions. This distinctive characteristic demonstrates their great promise for various optoelectronic and energy device applications.

## 2 Computational method

The calculations presented in this study were performed using the WIEN2k software package [27]. This package employs the full-potential linearized augmented plane wave (FP-LAPW) technique as a component of DFT theory [28]. In this context, the Kohn-Sham equation is solved by expanding the wave functions in spherical harmonics, which are formed within the atomic spheres. To describe the interstitial regions between atoms in the unit cell, the plane wave expansion technique was employed. An angular momentum value of l_max_ = 10 was utilized. The G_max_ parameter, set at 12.0 bohr^−1^, determined the maximum range of the expansion. The cutoff parameter, denoted as R_MT_ × K_max_, was set at 7, where R_MT_ represents the radius of the smallest muffin tin (atomic) sphere and K_max_ indicates the magnitude of the largest wave vector. The exchange-correlation (E_xc_) potential was treated by approximating the generalized gradient of Perdew-Burke-Enzerhof (GGA-PBE) [29]. The modified Becke-Johnson (TB-mBJ) approach is introduced to improve the accuracy of the calculations, particularly for the gap, and to closely approximate the experimental data [30,31]. The energy cutoff separating the core and valence states is set at −6.0 Ry. For the atoms Cs, In, Sb, F, and Cl, the respective muffin-tin radius spheres (R_MT_) were fixed at ${\;R}_{MT}^{Cs}={\;R}_{MT}^{In}={\;R}_{MT}^{Sb}$, ${\;R}_{MT}^{F}=$ 1.92, and ${\;R}_{MT}^{Cl}=$ 2.28 Bohr. Brillouin-zone (BZ) integrations within the self-consistency cycles are performed using 200 k-points spanning the entire BZ. Electronic and optical properties require denser meshes with higher accuracy. To achieve this, 1000 k-points are employed in the mesh generation process. The Gibbs2 software package is used to perform the calculations involving these dense meshes [32] and to compute the thermodynamic properties of Cs_2_InSbX_6_ (X = Cl, F). It relies on outputs from periodic quantum-mechanical simulations for solids, including the energy-volume relationship and, when applicable, the harmonic phonon frequencies. These simulations are carried out within the framework of the quasi-harmonic approximation.

## 3 Results and discussions

### 3.1 Structure analysis

**Fig. 1:**
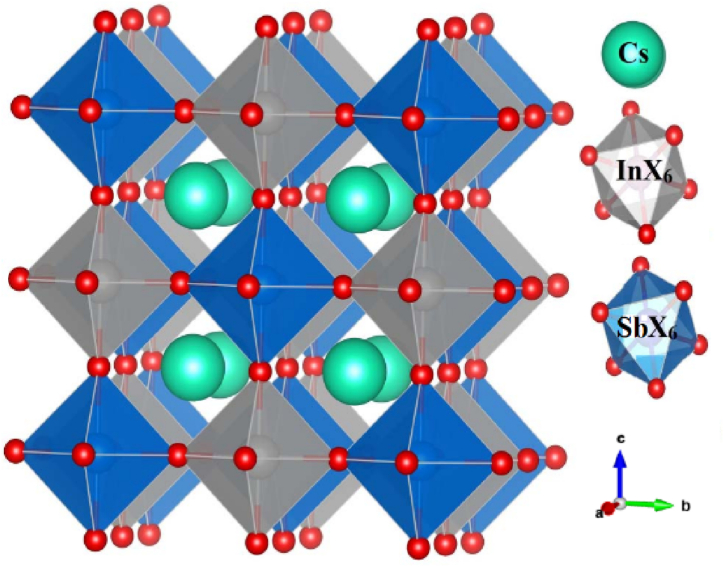
Crystal structure of Cs_2_InSbX_6_ (X = F, Cl).

**Fig. 2:**
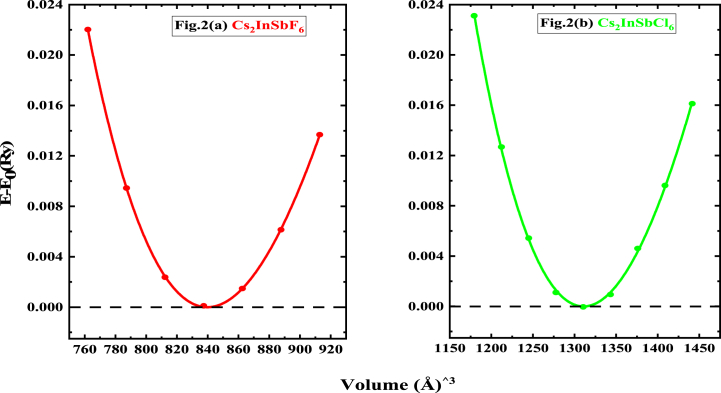
Variation of the difference between the system energy in the deformed state, *E*, and the equilibrium energy, *E*_0_, as function of volume.

Geometric optimization seeks to identify the atomic positions that correspond to the minimum energy state of the system for a particular configuration. This is achieved through an iterative process that involves adjusting the positions of atoms within the unit cell (Fig. 1) to minimize interatomic forces and attain structural equilibrium. In essence, the positions of the atoms are repeatedly modified until the forces acting on each atom approach zero, indicating that the interatomic forces are balanced and the system has reached its lowest possible energy state for that specific configuration. The optimized parameters for Cs_2_InSbX_6_ (X = F, Cl) are presented in Table 1, while Fig. 2 illustrates how the total system energy changes with the unit cell volume. By carrying out this crucial step, the optimal system parameters including the equilibrium volume, bulk modulus, and ground state energy, can be determined using the Birch-Murnaghan equation [33]. The lattice parameters of Cs_2_InSbX_6_ (X = F, Cl), derived through numerical simulation, show a remarkable agreement with the values reported in Refs. [22,23].

**Table 1:** Calculated tolerance factor τ (evaluated both via Eq. (1) and Eq. (2)), Octahedral factor, μ = *R*_B_/*R*_X_, Lattice constants a, Bulk modulus B, Bulk modulus derivative, B’, Equilibrium volume V_o_, and Equilibrium energy E_0_ of Cs_2_InSbX_6_ (X = F, Cl).

Compound	τ, Eq. 1	τ, Eq. 2	μ	a (Å)	B	B’	V_o_ (Å)^3^	E_0_ (Ry)
Cs_2_InSbF_6_	1.10	3.52	0.59	9.44	39.1165	5.4927	840.0999	−57094.488
				9.80^a^				
Cs_2_InSbCl_6_	0.95	4.13	0.43	10.94	21.7061	5.3512	1310.4438	−61434.026
				11.14^b^				

a Reference [34].

b Reference [35].

On the other hand, the lattice constant showed a consistent increase upon replacing the halogens, which can be attributed to the larger size of the Cl compared to F anions. In addition, various techniques were employed to examine the stability of these double perovskites Cs_2_InSbX_6_ (X = F, Cl). The ionic radii of the formulas A_2_BB’X_6_ were used to determine the Goldschmidt tolerance (τ) factor and octahedral (μ) factor [36].

(1) $$\tau_{G}=\frac{R_{A}+R_{X}}{\sqrt{2}\left(<R_{B^{\prime },B}>+R_{X}\right)}$$

Where R_X_ and R_A_ are the radii of the halogen atoms and Cs atom, respectively, $\left\langle R_{B^{\prime },B}\right\rangle$ are the average radii of sites B^+^ and B^3+^. The data obtained for the tolerance and octahedral factors are listed and compared with other theoretical results in Table 1. A value of μ in the range of 0,43 < μ < 0.59 and a value of ${\;\tau }_{G}$ in the range of 0.95 < ${\;\tau }_{G}$ < 1.10 were proposed. The present study reveals that Cs_2_InSbX_6_ (X = F, Cl) exhibits a perfect perovskite structure, as evidenced by the tolerance and octahedral factors. Specifically, the tolerance values fall within the range of 0.95–1.10, while the octahedral values are situated between 0.43 and 0.59. These findings suggest that the material crystal lattice is highly ordered, which is a crucial aspect of perovskite structure stability. Notably, the precision and probabilistic nature of the tolerance factor enable a deeper understanding of the physical properties that govern the formation and stability of various types of perovskites, including both organic and inorganic compounds. Furthermore, the development of a novel tolerance factor formula provides a valuable tool for predicting the stability of yet unknown perovskite materials, paving the way for further research and potential applications in this exciting field [37]:

(2) $$\tau_{G}=\frac{R_{X\;}}{\left\langle {\;R}_{B^{\prime },B}\right\rangle }-n_{A\;}\left[n_{A\;}-\frac{R_{A}≮{\;R}_{B^{\prime },B}\;>}{\ln\left(R_{A}≮{\;R}_{B^{\prime },B}>\right)}\;\right]$$

Where $n_{A}$ represent the A oxidation state. Table 1 lists the τ values calculated using Eq. (2) and clarifies that the values are below 4.13 (stable materials are considered when ${\;\tau }_{G}$ < 4.63) [37]. The stability of Cs_2_InSbX_6_ in cubic crystal structures was confirmed by the tolerance and octahedral factors, which were studied in the Fm-3m space group, corresponding to an ideal double perovskite.

To confirm the stability of the compounds Cs_2_InSbX_6_, where X varies from F to Cl, the calculations of their formation energies were carried out on the basis of the following relationship [38,39]:

$$\Delta E=\frac{E_{\mathrm{Tot}\;}-ƩnE_{a}}{N}$$

Where n is the number of atoms in the species, N is the number of atoms in a unit cell, and E_a_ is the energy of an individual atom in its ground state. The energy of an individual atom can also be determined by optimizing a separate atom. The estimated formation energies of Cs_2_InSbF_6_ and Cs_2_InSbCl_6_ are −14.45 eV, −15.86 eV, respectively. The compounds were thermodynamically stable, as indicated by their negative formation energies. Additionally, the phonon dispersions of similar compounds have recently been calculated by Aslam et al. [23], which further supports the stability of our material.

### 3.2 Electronic properties

**Fig. 3:**
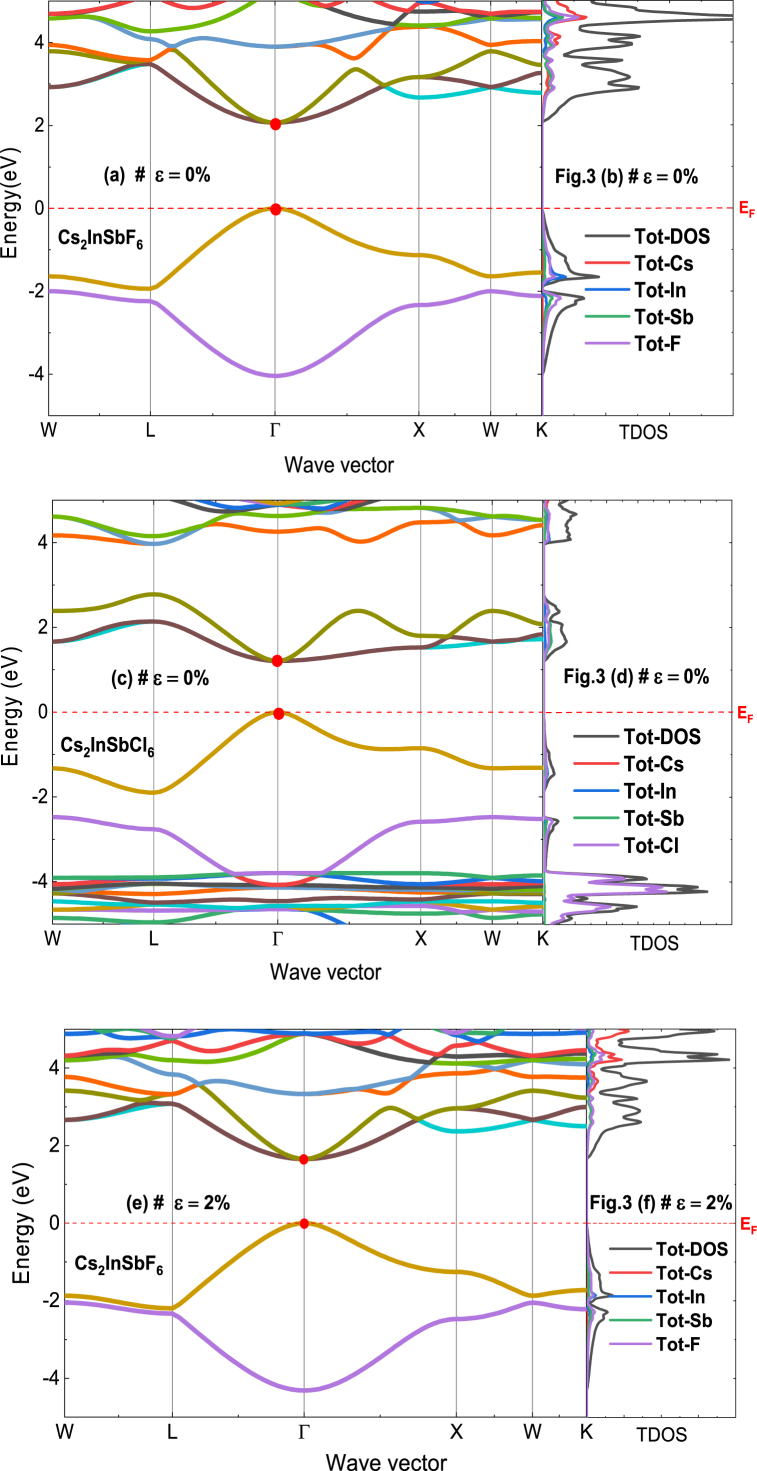

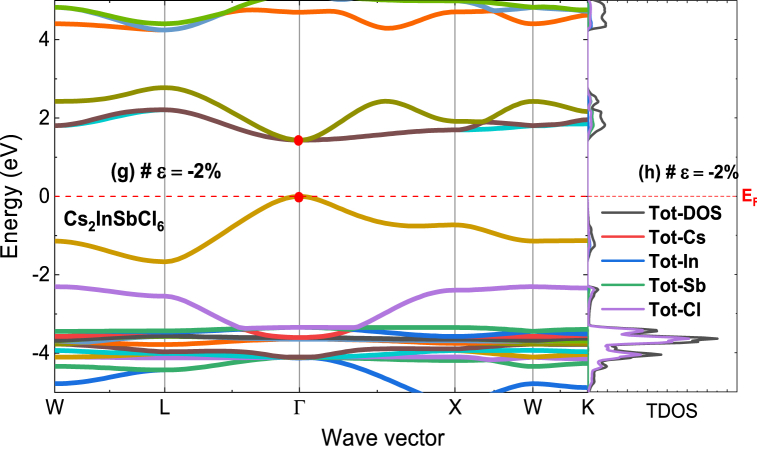
Band structure and total DOS of (a) Cs_2_InSbF_6_, and (b) Cs_2_InSbCl_6_ without strain. Band structure and total DOS of (e) Cs_2_InSbF_6_ with tensile strain of 2 %, and (g) Cs_2_InSbCl_6_ with compressive train of −2%.

In the following analysis, the electronic properties of Cs_2_InSbX_6_ (X = F, Cl) are studied under both strained and unstrained conditions using two distinct approximations: GGA-PBE and LSDA-mBJ (a combination of modified Becke-Johnson exchange potential and Local Spin Density Approximation). This is done to gain insight into how strain affects the electronic structure of the material. The goal is to identify the impact of LSDA-mBJ approximation on the electronic properties. Fig. 3 presents the computed density of states (DOS) and band structures for Cs_2_InSbX_6_ (X = F, Cl). Looking at the orbital characteristics, one can see that the conduction band is mainly from contributions of the In-5s, Sb-6s, Sb-6p and X-3p, 4p and 5p orbitals. Whereas the valence band is formed by the overlapping orbitals of the In-5p, Sb-6p and X-3p, 4p and 5p states. The band gap of undeformed Cs₂InSbX₆ (X = F, Cl) is 2.06 eV for Cs₂InSbF₆ and 1.20 eV for Cs₂InSbCl₆, consistent with the band gap values of comparable compounds presented in Table .1. The increase in the band gap under negative strain can be attributed to the elongation of atomic bonds within the material. As the negative strain (compressive stress) increases, it results in a greater distance between atoms, which subsequently reduces orbital overlap and leads to a widening of the band gap. This effect is consistent with the behavior observed in materials such as Cs₃Bi₂I₉ and Cs₂AgBi₂I₉ [40], and PbBi_2_Se_4_ [41], where the influence of strain on the band gap has been extensively studied. These references highlight how the structural distortions induced by strain can affect electronic properties, including both band gap reduction and enlargement, depending on the nature of the strain applied. The band structure of Cs_2_InSbX_6_ (X = F, Cl) is further characterized by analyzing its electronic properties at high-symmetry points and lines in the first Brillouin zone, corresponding to the orthorhombic lattice. The investigation focuses on six specific points: W, L, Γ, X, W, and K, using the LSDA-mBJ method as shown in Fig. 3.

**Fig. 4:**
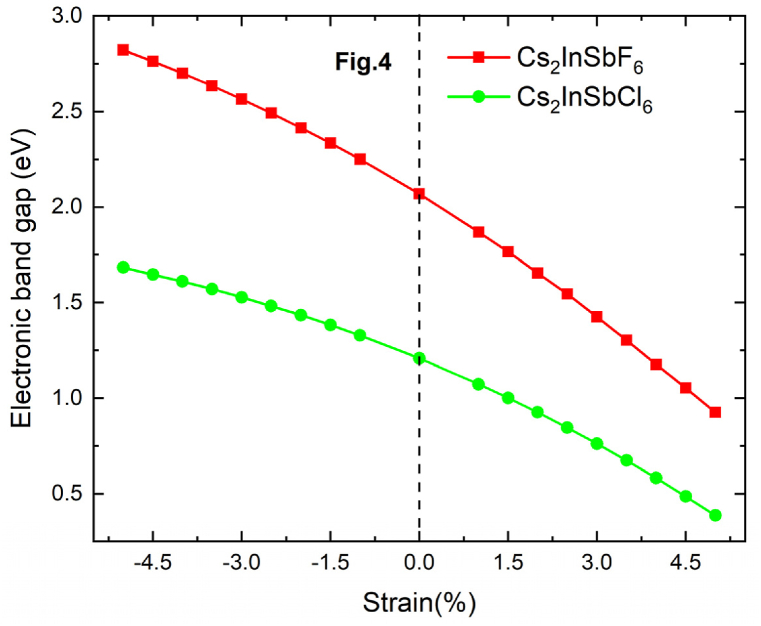
Variation of electronic band gap versus compressive (negative) and tensile (positive) strain.

The strain applied to Cs_2_InSbX_6_ (X = F, Cl) was achieved by modifying the volume of its unit cell using the following equation $\varepsilon \left(\%\right)=\left(a-a_{0}\right)/a_{0}$ × 100 [42,43]. The applied constraints were carried out independently on time. For each percentage, we calculated the new lattice parameter, afterward we investigated the properties. The strain (*ε*) was determined as a percentage change in the lattice parameter relative to the unstrained cell parameter $a_{0}$. The effect of applied strain on the band gap energy was significant, exhibiting a linear behavior of $E_{g}$ (ε). Fig. 4 displays the variation in gap energy versus the strain for both compressive and tensile strain. Simulations were performed to investigate the impact of compressive strain on the band gap energy in the cubic structure of these compounds. The simulations were carried out under compressive strain along the three crystallographic directions (a, b, and c) simultaneously, covering a range of −1% to −5%. This allowed for a comprehensive understanding of the effects of strain on the band gap energy. The band gap energy was estimated in their unstrained state to be 2.06 eV and 1.20 eV for Cs_2_InSbF_6_ and Cs_2_InSbCl_6_, respectively. Under the compressive strain, the band gap energy decreased linearly with the percentage of strain. Its starting value is of 2.8 eV for Cs_2_InSbF_6_ and 1.7 eV for Cs_2_InSbCl_6_, at a strain of −5%. This consistent linear increase in the band gap can be attributed to the compression-induced effects on the valence band maximum (VBM) and conduction band minimum (CBM). Tensile strain simulations were also performed in the range from 1 % to 5 %, during which the band gap energy decreased linearly to 1.02 eV for Cs_2_InSbF_6_ and 0.38 eV for Cs_2_InSbCl_6_ at a strain of 5 %.

**Fig. 5:**
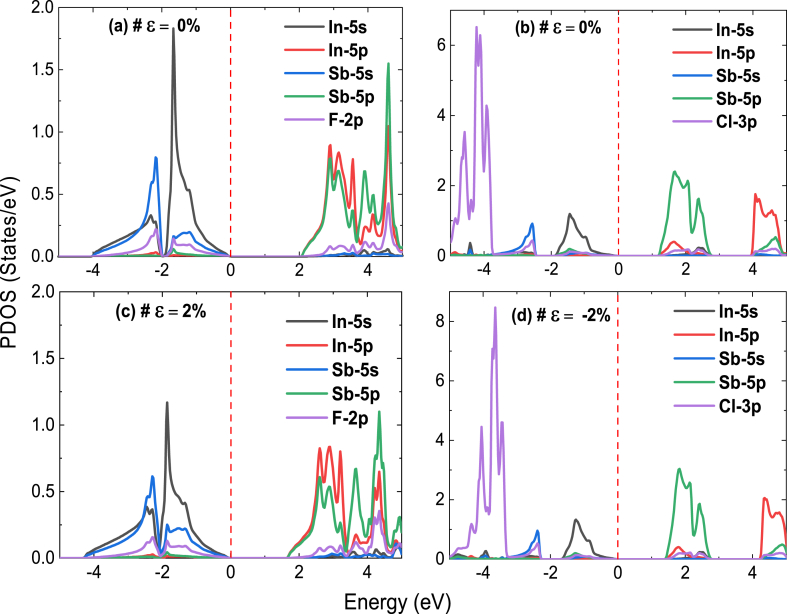
Partial DOS of (a) Cs_2_InSbF_6_, and (b) Cs_2_InSbCl_6_ without strain, (c) with tensile strain of 2 %, and (d) with compressive train of −2%.

For photovoltaic applications, a band gap energy of approximately 1.1–1.5 eV is often aimed. This range of band gaps enables efficient absorption of solar light in the visible spectrum region, where most of the solar energy is concentrated. A material with a band gap within this range can generate photoelectrons upon light absorption, contributing to the conversion of sunlight into electrical energy. Thus, it is essential to tailor the material properties through various modifications to achieve the adequate band gap value. In particular, applying strains of ±2 % leads to the following results: 1.66 eV for Cs_2_InSbF_6_ (*ε* = 2 %) and 1.44 eV for Cs_2_InSbF_6_ (*ε* = −2%). By adjusting the material structure, the electronic energy levels can be modulated (as shown in Fig. 3, Fig. 5), allowing to attain the desired energy gap for a given application. Choice of ±2 % strain was considered to investigate the effects of moderate strain on the Cs_2_InSbX_6_ compound, as they are optimal for achieving the aimed energy gap range for photovoltaic applications. The modification of atomic orbital positions can result in changes to the electronic energy levels associated with them, which in turn affects the partial density of states (DOS). This, therefore, causes shifts in the peaks of electronic states related to those orbitals, ultimately influencing the gap energy.

This extensive bandwidth spread leads to very relatively effective masses for carriers [31]. From the parabolic fitting of the band edges, the effective electron masses $m_{e}^{*}$ and holes (cavities) $m_{h}^{*}$, as a function of m_0_, were calculated using the following formula:

(3) $$m^{*}=\hbar^{2}{\left[\frac{\partial^{2}E\left(k\right)}{\partial k^{2}}\right]}^{-1}$$

Where E(k) and k represent the band-edge eigenvalues and wavevectors, respectively. Table 2 displays the calculated effective carrier masses for Cs_2_InSbX_6_ (X = F, Cl) with and without constraints. The obtained values of ${\;m}_{e}^{*}$ and $m_{h}^{*}$ agreed well with those reported in the literature [30]. The effective masses were also compared to those of MAPbI_3_. In the unconstrained case (0 %), for Cs_2_InSbF_6_, the effective mass ${\;m}_{e}^{*}$ is 0.78, indicating relatively high electron mobility and suggesting good electronic performance.

**Table 2:** Calculated Bond Lengths $r_{ij}$, Band Gap $E_{g}$, and Effective Masses of Electrons $m_{e}^{*}$ and Holes $m_{h}^{*}$ ($m_{0}$) versus compressive and tensile stress of Cs_2_InSbX_6_ (X = F, Cl).

Parameters		Cs_2_InSbF_6_	Cs_2_InSbCl_6_	MAPbI_3_
$E_{g}$ (eV)	ε = 0 %	2.06	1.20	
		2.01^d^	1.02^a^^,^^b^ 1.37^d^	
	ε = 2 %	1.66	0.93	
	ε = −2%	2.41	1.44	
$m_{e}^{*}/m_{0}$	ε = 0 %	0.78	0.73	0.19^c^
	ε = 2 %	0.74	0.61	
	ε = −2%	0.82	0.76	
$m_{h}^{*\;}/m_{0}$	ε = 0 %	0.44	0.23	0.25^c^
	ε = 2 %	0.13	0.17	
	ε = −2%	0.40	0.23	
			0.25^b^	
$r_{ij}$ (Å)	Sb-X (x_6_)	2.09	2.57	
	In-X (x_6_)	2.13	2.61	
	Cs-X (x_12_)	3.00	3.48	

a Reference [43].

b Reference [22].

c Reference [3].

d Reference [44].

The hole mass $m_{h}^{*}$ for Cs_2_InSbF_6_ is 0.44, signifying moderate hole mobility. For Cs_2_InSbCl_6_, the effective mass ${\;m}_{e}^{*}$ is 0.73, also showing decent electron mobility. The hole mass $m_{h}^{*}$ for Cs_2_InSbCl_6_ is 0.23, indicating lower hole mobility compared to Cs_2_InSbF_6_. When applying a (−2%) tensile strain to Cs_2_InSbF_6_, the effective mass ${\;m}_{e}^{*}$ increases to 0.82, indicating a reduction in electron mobility under tensile strain. The hole mass $m_{h}^{*}$ also increases to 0.40, suggesting improved hole mobility but still lower than the unconstrained configuration. For Cs_2_InSbCl_6_ under (−2%) tensile strain, the effective mass ${\;m}_{e}^{*}$ increases to 0.76, indicating a slight reduction in electron mobility. The hole mass $m_{h}^{*}$ remains relatively stable at 0.23, showing hole mobility similar to the unconstrained configuration. In the case of a (2 %) compression applied to Cs_2_InSbF_6_, the effective mass ${\;m}_{e}^{*}$ slightly decreases to 0.74, suggesting that electron mobility remains reasonable despite the strain. The hole mass $m_{h}^{*}$ decreases significantly to 0.13, indicating a substantial improvement in hole mobility. For Cs_2_InSbCl_6_ under (2 %) compression, the effective mass ${\;m}_{e}^{*}$ significantly decreases to 0.61, indicating reduced electron mobility under strain. The hole mass $m_{h}^{*}$ reveals a slight increase to 0.17, indicating that the heavy hole mass induces a decrease in the hole mobility compared to the non-strained case.

### 3.3 Optical properties

In this section, the effect of strain on the optical properties of Cs_2_InSbX_6_ (X = F, Cl) was examined by calculating the dielectric function, *ε*(ω), which is a complex quantity that describes how the material interacts with electromagnetic waves. The dielectric function is a crucial parameter in determining the optical properties of a material, such as its refractive index, absorption coefficient, and reflectivity. By analyzing the variation of these properties, researchers can gain insights into how the application of mechanical stress affects the optical properties of Cs_2_InSbX_6_. For example, they may observe changes in the refractive index, absorption coefficients, or other optical constants, which can be used to understand the mechanisms underlying the material optical behavior. Furthermore, these insights can be used to optimize the performance of optoelectronic devices that rely on Cs_2_InSbX_6_, such as photodetectors, solar cells, or LEDs. By engineering the material properties through careful control of strain, researchers can enhance device efficiency, speed, or sensitivity, ultimately leading to improved performance and functionality.

To calculate the dielectric constant of Cs_2_InSbX_6_, we employed the OPTIC program in Wien2k to calculate the frequency-dependent dielectric function ε(ω), which includes the real part $\varepsilon_{1}\left(\omega \right)$ and imaginary part $\varepsilon_{2}\left(\omega \right)$. The OPTIC module computes the imaginary part of the dielectric function by summing over the transitions between occupied and unoccupied states, using the momentum matrix elements between these states. The Drude model represents ε(ω) as the sum of two components, ($\varepsilon_{1}\left(\omega \right)+{i\varepsilon }_{2}\left(\omega \right))$ [45,46], where $\varepsilon_{2}\left(\omega \right)$ is the imaginary part. When a material absorbs electromagnetic radiation, its electrons are excited to higher energy states, forming electron-hole pairs and leading to energy loss in the form of heat. The imaginary part of the dielectric function, $\varepsilon_{2}\left(\omega \right)$, is directly related to the material absorption. On the other hand, $\varepsilon_{1}\left(\omega \right)$ represents the real part of ε(ω) and is associated with the material polarization in response to an external electric field [46,47]. When an electric field is applied, the material electrons are displaced, leading to charge separation and the creation of an electric dipole moment. The magnitude of this dipole moment depends on the real part of the dielectric function, which characterizes the dielectric properties of the material. The real and imaginary parts of the dielectric function can be computed using the Kramer-Kronig relations. Specifically, $\varepsilon_{1}\left(\omega \right)$ is obtained from an integral involving $\varepsilon_{2}\left(\omega \right)$, while $\varepsilon_{2}\left(\omega \right)$ is derived using a summation over relevant quantum states:

(4) $$\varepsilon_{1}\left(\omega \right)=1+\frac{2}{\pi }\int_{0}^{\infty }\frac{\varepsilon_{2}\left(\omega^{\prime }\right)\omega^{\prime }\mathrm{d}\omega^{\prime }}{{\omega^{\prime }}^{2}-\omega^{2}}$$

(5) $$\varepsilon_{2}\left(\omega \right)=\frac{{4\pi }^{2}e^{2}}{m^{2}\omega^{2}}\sum_{ij}|<i\left|M\right|f>{|}^{2}.\left(f_{i}\left(1-f_{i}\right)\right)\delta \left(E_{f}-E_{i}-\hbar \omega \right)d^{3}k$$

The material ability to conduct electricity under an external electric field at a specific frequency is characterized by the optical conductivity, denoted as σ(ω). Maxwell equations allow to calculate σ(ω) based on the dielectric constant ε(ω) according to the formula:

(6) $$\varepsilon \left(\omega \right)=\varepsilon_{\infty }+i\frac{\sigma \left(\omega \right)}{\omega \varepsilon_{0}}$$

With $\varepsilon_{\infty }$ representing the relative permittivity and $\varepsilon_{0}$ the permittivity of vacuum. Manipulating the equation of electromagnetic wave propagation within a solid reveal that the refractive index is a complex number, given as ${\tilde{n}}^{2}={\left(n\left(\omega \right)+ik\left(\omega \right)\right)}^{2}=\varepsilon \left(\omega \right)$. The real part of the refractive index, n(ω), indicates how much the velocity of light slows down as it traverses the material. Meanwhile, the imaginary part, k(ω), indicates the light absorption capability in the material, often referred to as the extinction coefficient. These physical parameters, n(ω) and k(ω), are related to the dielectric function components $\varepsilon_{1}\left(\omega \right)$ and $\varepsilon_{2}\left(\omega \right)$ through mathematical expressions [23,46]:

(7) $$n\left(\omega \right)=\frac{1}{\sqrt{2}}{\left(\sqrt{\varepsilon_{1}{\left(\omega \right)}^{2}+\varepsilon_{2}{\left(\omega \right)}^{2}}+\varepsilon_{1}{\left(\omega \right)}^{2}\right)}^{1/2}$$

(8) $$k\left(\omega \right)=\frac{1}{\sqrt{2}}{\left(\sqrt{\varepsilon_{1}{\left(\omega \right)}^{2}+\varepsilon_{2}{\left(\omega \right)}^{2}}-\varepsilon_{1}{\left(\omega \right)}^{2}\right)}^{1/2}$$

The real and imaginary parts of the refractive index provide essential information about the optical properties, including its ability to reflect light (optical reflectivity R(ω)) and its absorption coefficient, α(ω), which can be determined from k(ω) and relates to the rate of light absorption at a given frequency as follows [47]:

(9) $$R\left(\omega \right)=\frac{{\left(n\left(\omega \right)-1\right))}^{2}+k{\left(\omega \right)}^{2}}{{\left(n\left(\omega \right)+1\right))}^{2}+k{\left(\omega \right)}^{2}}$$

(10) $$\alpha \left(\omega \right)=\frac{\omega }{c}k\left(\omega \right)$$

The variation in optical properties under strain offers valuable insights into the coupling between mechanical and optical behaviors of materials, which has far-reaching implications for various fields, including optoelectronics and photonics. This phenomenon holds significant interest in materials science and engineering, as it unveils how the optical response of a material can be tailored or controlled by mechanical means. When subjected to strain, a material crystal lattice experiences alterations in bond lengths, angles, and arrangement, influencing the behavior of both electrons and photons within the material. These changes induce variations in optical properties, providing a means to manipulate them through mechanical manipulation. For this purpose, ab-initio calculations were used to predict the effect of strains on the optical properties of the double perovskite Cs_2_InSbX_6_.

**Fig. 6:**
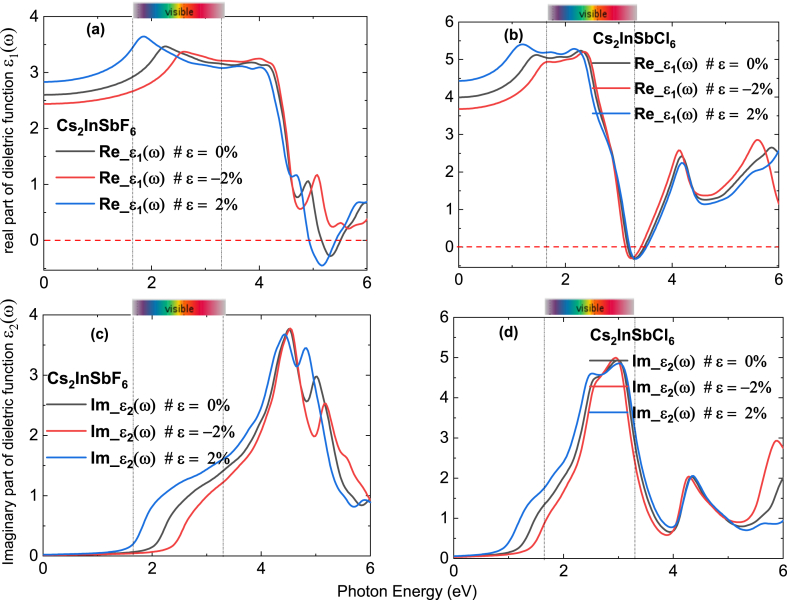
(a)–(b) Real and **(c)–(d)** Imaginary part of dielectric function of Cs_2_InSbF_6_, and Cs_2_InSbCl_6_ without strain 0 %, with tensile strain of 2 %, and with compressive train of −2%.

Fig. 6 shows the dielectric function of the compound with and without strain. In Fig. 6a and 6b, the real part $\varepsilon_{1}\left(\omega \right)$ of Cs_2_InSbF_6_ and Cs_2_InSbCl_6_ depends significantly on applied strain, where the zero-frequency dielectric function $\varepsilon_{1}\left(0\right)$ increases from the unstrained value (2.6) to the value 2.7 for Cs_2_InSbF_6_ and to 4.5 for Cs_2_InSbCl_6_ when strains are 2 %, whereas it decreases to 2.4 when compressive strains of −2% are applied for Cs_2_InSbF_6_ and 3.6 for Cs_2_InSbCl_6_. This change arises from the fact that mechanical strain causes a modification in the spacing between electric charges or dipoles, which can influence the dielectric constant. Moreover, $\varepsilon_{1}\left(\omega \right)$ increases in the visible spectrum at strains of 2 % for the Cs_2_InSbF_6_. As depicted in Fig. 6c, and 6.d the imaginary part of the dielectric function illustrates the robust capacity of Cs_2_InSbF_6_ and Cs_2_InSbCl_6_ to preserve absorbed energy, underscoring its suitability as an ideal material for absorbing light in solar cell applications.

**Fig. 7:**
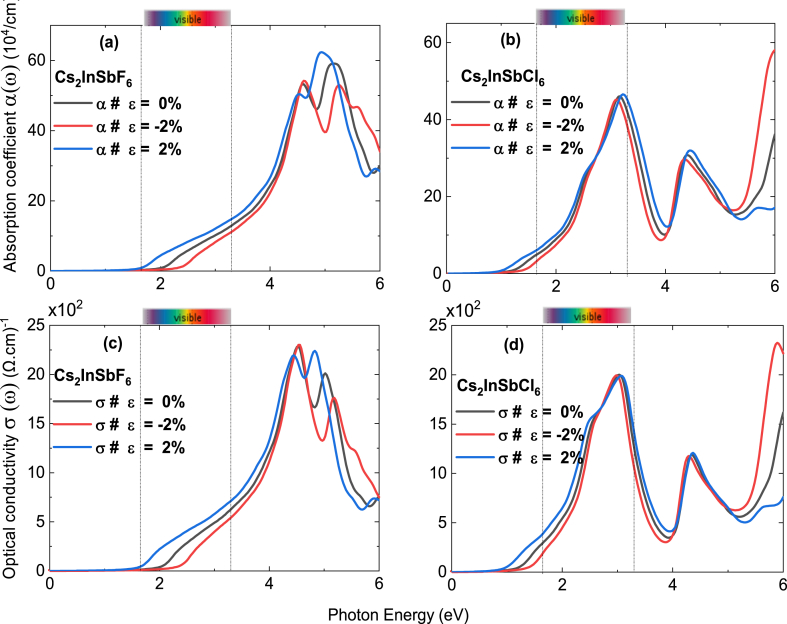
(a)–(b) Absorption coefficient and **(c)–(d)** Optical conductivity of Cs_2_InSbF_6_, and Cs_2_InSbCl_6_ without strain 0 %, with tensile strain of 2 %, and with compressive train of −2%.

Fig. 7a and 7b shows the response of the absorption coefficient of Cs_2_InSbX_6_ for various values of photon energy. Clearly evident is the robust visible spectrum absorption of Cs_2_InSbCl_6_ (4 × 10^5^/cm at 3 eV energy in contrast to Cs_2_InSbF_6_, which has low absorption in the visible spectrum, which improves with mechanical strain. When considering technological applications, the quest for the optimal range of optical conductivity becomes significant. In both compounds, a peak conductivity of approximately 2 (kΩ cm)^−1^ is detected (Fig. 7c and 7d). This occurs within the visible spectrum for Cs_2_InSbCl_6_ and within the ultraviolet spectrum for Cs_2_InSbF_6_.

**Fig. 8:**
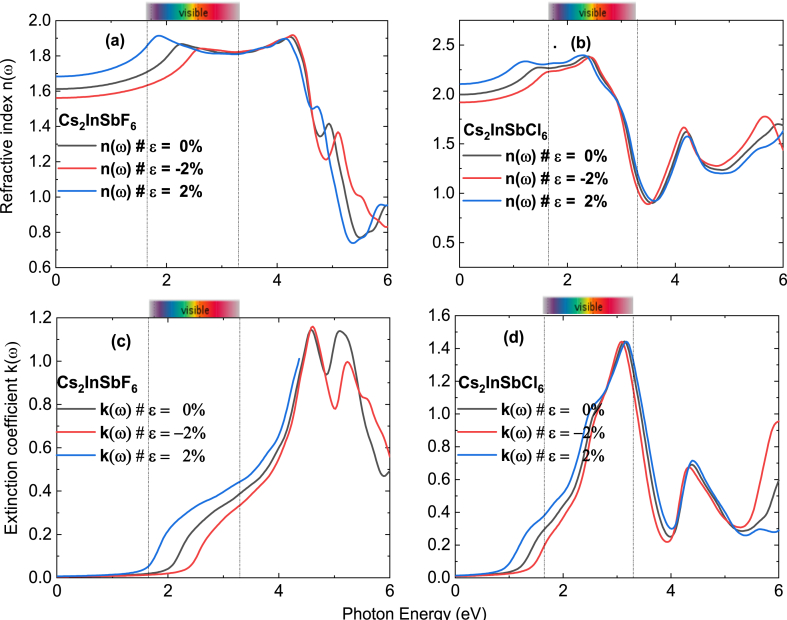
(a)–(b) Refractive index (**c)–(d)** Extinction coefficient of Cs_2_InSbF_6_, and Cs_2_InSbCl_6_ without strain 0 %, with tensile strain of 2 %, and with compressive train of −2%.

Fig. 8 illustrates the refractive index and extinction coefficient as functions of energy for Cs_2_InSbX_6_. The static refractive index for Cs_2_InSbF_6_ is n(0) = 1.6, and n (0) = 2.0 for Cs_2_InSbCl_6_ (unconstrained). In the visible spectrum, the value shifts to 1.9 for Cs_2_InSbF_6_ and reaches approximately 2.25 for Cs_2_InSbCl_6_ at a photon energy of 2 eV. This characteristic renders Cs_2_InSbCl_6_ exceptionally valuable for photovoltaic applications. The refractive index demonstrates significant sensitivity to deformation, exhibiting an increase under a deformation of *ε* = 2 % for both compounds. The extinction coefficient of studied compounds under both deformation cases aligns with the imaginary component of the dielectric constant $\varepsilon_{2}$ (ω), signifying electronic transitions at energy levels corresponding to peaks, namely 1.4 for Cs_2_InSbCl_6_ at around 3 eV, and approximately 1.1 for Cs_2_InSbF_6_ at around 4.5 eV. The optical characteristics of Cs_2_InSbF_6_ demonstrate its potential as an absorbing layer, exhibiting a desirable $E_{g}$ value and robust light absorption capabilities. Particularly noteworthy are its enhanced absorption and rapid photo-response, which make it an attractive candidate for photovoltaic applications and justify further exploration. A significant discovery arises from applying a 2 % strain along the X-axis, demonstrating substantial interaction between light and the material. This interaction has led to a notable increase in the optical properties of the material. This finding holds paramount importance as it opens promising avenues for targeted improvement of materials, particularly in the field of optoelectronics and beyond. This advancement suggests that strain-induced modifications can be strategically exploited to optimize material performance in various applications, thereby providing new opportunities for engineering more efficient and light-sensitive devices.

### 3.4 Photocatalytic properties

#### 3.4.1 Photocatalytic activity

The photocatalytic process relies on the capacity of a semiconductor material to react to light stimulation. When a photon with sufficient energy (greater than the 1.23 eV bandgap) interacts with the semiconductor, it triggers the formation of an electron-hole pair. This occurs because the absorbed photon excites an electron from the valence band into the conduction band, leaving behind a hole in the valence band. Then, the energized electron migrates through the conduction band, contributing to various chemical reactions that facilitate the degradation or transformation of organic pollutants. This procedure provides a redox process to decompose H_2_O. Strains can be effective solutions for affecting the bandgap, the arrangement of the band edges and the type of electronic transition.

In our work, we have studied the impact of strain on the photocatalytic activity of the antimony-based materials Cs_2_InSbX_6_ (X = F, Cl). This analysis focuses on the analysis of the positions of the valence and conduction bands in the oxidation-reduction diagram with the investigation of the effective rate of strain for good management of the structure of a material and its optimal activity in photocatalysis.

To effectively manipulate the engineering of a gap and its alignment, we use the principles of semiconductor electronegativity and the Nernst equation (as shown in Table 3) to determine the locations of the valence and conduction band edges. By employing these concepts, we can calculate the position of the edge of the conduction band ($E_{CB}$) using the following formula:

(11) $$E_{CB}=\chi \left(S\right)-E_{0}-1/2E_{g}$$

where χ (S) represents the Fermi level of the semiconductor, $E_{0}$ is the scale factor connecting the redox level of the reference electrode to the absolute vacuum scale (typically around 4.5 eV for a standard hydrogen electrode), and $E_{g}$ is the band gap itself. Additionally, we can find the position of the valence band edge ($E_{VB}$) by adding the band gap ($E_{g}$) to the conduction band edge ($E_{CB}$):

(12) $$E_{VB}=E_{CB}+E_{g}$$

**Table 3:** Band gap ($E_{g}$), electronegativity (χ), relative energy of valence band edge ($E_{VB}$) and Energy levels of conduction band edge ($E_{CB}$).

Semiconductors	χ (eV)	Strain (*ε*)	$E_{g}$ (eV)	$E_{CB}$ (eV)	$E_{VB}$ (eV)
Cs_2_InSbF_6_	6.23	+5 %	0.926	1.267	2.193
		+4 %	1.177	1.141	2.318
		+3 %	1.426	1.017	2.443
		+2 %	1.654	0.903	2.557
		+1 %	1.869	0.795	2.664
		0 %	2.069	0.700	2.769
		−1%	2.250	0.605	2.855
		−2%	2.410	0.525	2.935
		−3%	2.564	0.448	3.012
		−4%	2.700	0.380	3.080
		−5%	2.821	0.319	3.140
Cs_2_InSbCl_6_	5.42	+5 %	0.386	0.727	1.113
		+4 %	0.581	0.629	1.210
		+3 %	0.762	0.539	1.301
		+2 %	0.930	0.455	1.385
		+1 %	1.073	0.383	1.456
		0 %	1.209	0.315	1.524
		−1%	1.328	0.256	1.584
		−2%	1.440	0.200	1.640
		−3%	1.529	0.155	1.684
		−4%	1.612	0.114	1.726
		−5%	1.684	0.078	1.762

By understanding the relationship between these parameters, we can better control the engineering of gaps and their arrangements in semiconductor materials [48,49]. χ is the electronegativity of semiconductor and it was calculated by the following equation [50,51]:

(13) $$\chi ={\left[{\chi \left(A\right)}^{a}{\chi \left(B\right)}^{b}{\chi \left(C\right)}^{c}\right]}^{1/\left(a+b+c\right)}$$

Where χ (A), χ (B), and χ (C) are the absolute electronegativities of atoms A, B and C, respectively. The average electronegativity of Cs_2_InSbX_6_ is 6.23 and 5.42 for Cs_2_InSbF_6_ and Cs_2_InSbCl_6_, respectively.

**Fig. 9.a:**
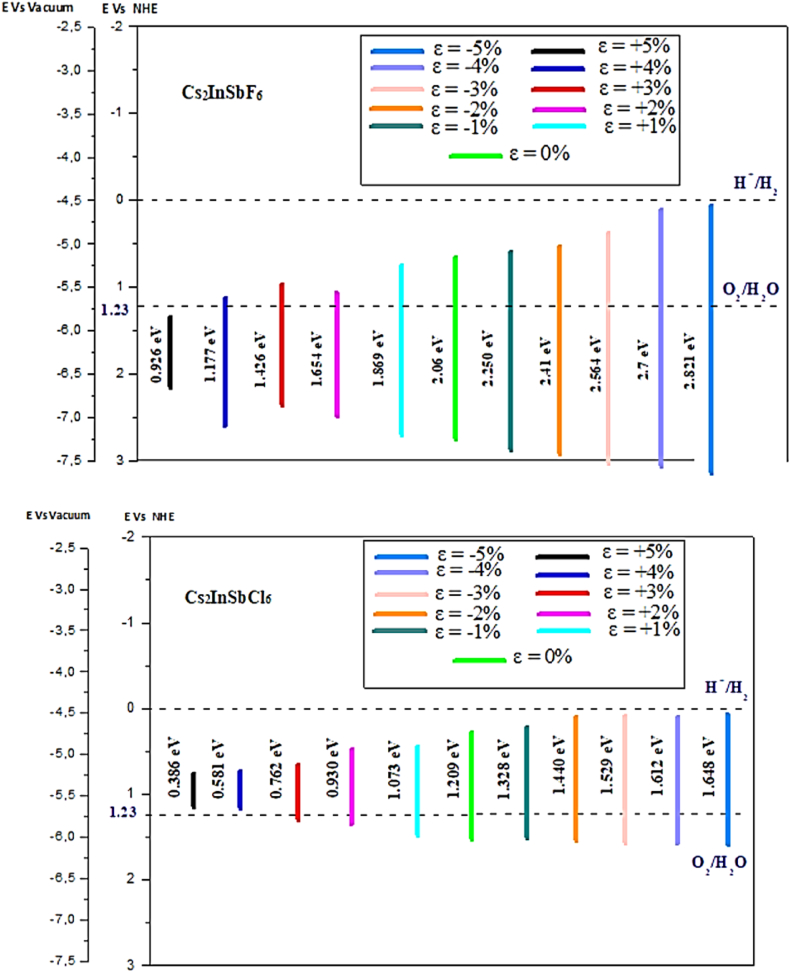

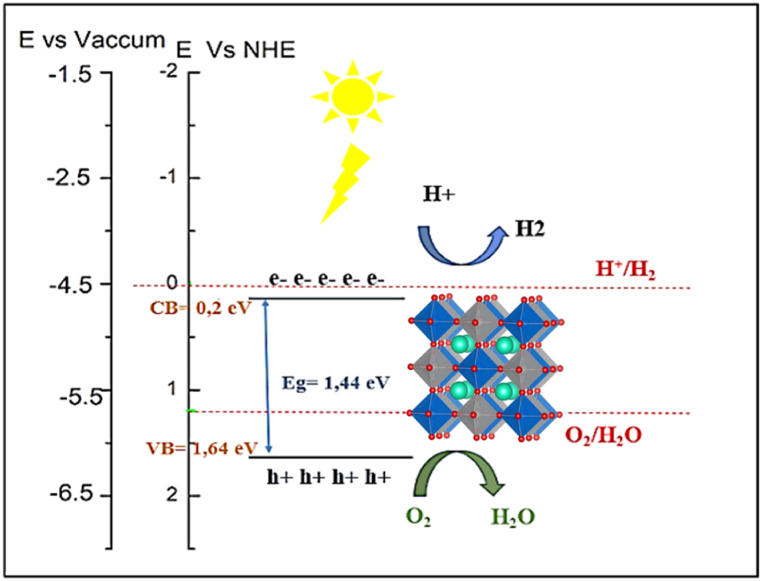
Schematic representation of the valence band, conduction band potentials and band gap energies of Cs_2_InSbX_6_ (X = F, Cl) Fig. 9b: Possible mechanisms of photocatalysis of Cs_2_InSbCl_6_.

Fig. 9a shows the arrangement of various structures based on applied strains. We have plotted the valence and conduction bands of Cs_2_InSbX_6_ (X = F, Cl) on to the photocatalysis phase diagram for analysis of suitable structures for photocatalytic activity. All structures of Cs_2_InSbF_6_ fall outside the photocatalysis active zone, having improper positions for photocatalytic activity and a widening energy gap despite stress. Conversely, Cs_2_InSbCl_6_ represents promising structures within the photocatalytic activity zone when subjected to tensile strain levels of *ε* = −2, −3, −4 and −5%, respectively. These strains enable a reduction in the energy gap, namely 1.440 eV, 1.529 eV, 1.612 eV, and 1.684 eV, with potential oxidation-reduction reactions possible under visible light due to probable action in the visible spectrum range. Our findings suggest that these distortions (−2%, −3%, −4%, −5%) may allow a band gaps reduction, leading to adequate reductions in photocatalytic activity while providing broad absorption spectra within the visible light domain and enabling water splitting.

The Cs_2_InSbCl_6_ system based on the *ε* = −2% order stress is the most accurate in promoting photocatalytic application. This mechanism focuses on the absorption of a photon from an energy source that causes the active electron ($e_{cb}^{-}$) to move to the conduction band, producing a positive hole ($h_{Vb}^{+}$) in the valence band (Fig. 9b):

$$\text{Light}\;\text{absorption}:{\text{Cs}}_{2}{\text{InSbCl}}_{6}+h\nu \;\to \;{{\text{Cs}}_{2}\text{InSb}{\text{Cl}}_{6}(e}_{cb}^{-})+{\text{Cs}}_{2}\text{InSb}{\text{Cl}}_{6}\left(h_{Vb}^{+}\right)$$

$$\text{Oxidation}\;\text{reaction}:2\;H_{2}O+4h_{Vb}^{+}\;\to \;O_{2}+4\;H^{+}$$

$$\text{Reduction}\;\text{reaction}:2\;H^{+}+2\;e_{cb}^{-}\;\to \;H_{2}$$

$$\text{Overall}\;\text{water}\;\text{splitting}:2\;H_{2}O\;\to \;2H_{2}+O_{2}$$

Photocatalysis is a promising solution that uses the ability of semiconductor materials to absorb light to produce chemical reactions in various fields.

The relationship between the optical properties and the structure of the electronic bands makes it possible to highlight the light absorption characteristics, which are based on the energy of the band gap and the absorption coefficient. These characteristics are one of the critical factors affecting photocatalytic power and activity.

We have highlighted the role of optical properties in photocatalytic activity, and this action has enabled us to compare the two structures and choose the most promising structure for photocatalytic power, which is Cs_2_InSbCl_6._

#### 3.4.2 Quantum efficiency

The application of mechanical stress to a material (strain) can significantly influence QE through various microscopic processes. It can significantly affect quantum efficiency (QE) by directly influencing bandgap by shifting absorption spectrum towards the optimal wavelength, carrier dynamics by enhancing carrier mobility, and material properties like structural distortion or defect formation. Also, it can create localized states that trap carriers and prevent non-radiative recombination. So, careful control of strain can be used to optimize the performance of various optoelectronic devices, such as solar cells, LEDs, and photodetectors.

The optical properties of Cs_2_InSbX_6_ (X = F, Cl) materials show strong absorption of solar radiation in the ultraviolet (UV) and solar visible (Vis) regions (see Fig. 7(a)). By focusing on their visible light absorption capacity, we can promote their energy production and water splitting capacity. Their optical characteristics have led us to calculate their quantum efficiency.

.QE is a critical parameter for evaluating the performance of materials in applications like photocatalysis, photovoltaics, and other solar technologies. It quantifies the efficiency with which a material converts incident photons into useable energy carriers, such as electrons or holes. This quantum efficiency is calculated using the following relationship [52]:

(14) $$\text{QE}=\left(1-R\right)\left({1-e}^{\alpha t}\right)$$

**Fig. 10:**
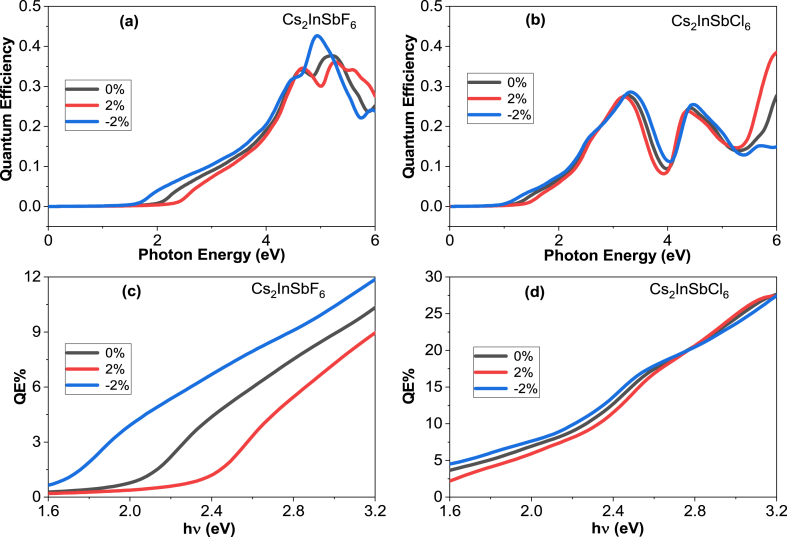
Quantum efficiency of Cs_2_InSbX_6_ (X = F, Cl) with different strain −2, 0 and 2.

Where R is the reflectivity and α is the absorption coefficient. The parameter t is the thickness of Cs_2_InSbX_6_ (X = F, Cl) sample. Effective absorption in the visible spectra and good optical performance enable efficient photocatalytic applications. The effective responses of Cs_2_InSbX_6_ (X = F, Cl) in QE, on the visible region, are represented in the range from 1.6 eV to 3.2 eV (Fig. 10).

For Cs_2_InSbF_6_, the optimum absorption in the visible range is in the order of 0.11 % at 3.1 eV for strain −2% and 0, while for strain 2 % is in the order of 0.08 %. For Cs_2_InSbCl_6_, the effective visible light absorption responses are of the order of 0.28 % at 3.1 eV for the strain with −2% while for the strain 2 % and 0 are of the order of 0.26 %. The strain −2% allows numerous more transitions to be obtained, which increases absorption induced by quantum efficiency increase.

## 4 Conclusion

This study demonstrates the potential of Cs₂InSbX₆ (X = F, Cl) as eco-friendly alternatives to lead-based perovskites in optoelectronic and photocatalytic applications. Using Density Functional Theory (DFT) calculations, we found that strain significantly affects their electronic and optical properties. For Cs₂InSbF₆, negative strain reduces the bandgap, enhancing its ability to absorb light and making it suitable for visible-light-driven photocatalysis. Conversely, Cs₂InSbCl₆ exhibits excellent visible light absorption and a high refractive index, making it ideal for solar cell applications. Our results show that applying mechanical strain can optimize these materials for different optoelectronic functions. Particularly, Cs₂InSbCl₆ under negative strain is a strong candidate for use in both solar energy harvesting and photocatalytic water splitting, offering a stable and environmentally sustainable alternative to traditional lead-based perovskites.

## CRediT authorship contribution statement

**S. Tariq:** Writing – original draft, Visualization, Investigation, Formal analysis, Conceptualization. **L.H. Omari:** Writing – review & editing, Writing – original draft, Validation, Supervision, Investigation, Formal analysis, Conceptualization. **F. Mezzat:** Writing – review & editing, Visualization, Software, Methodology, Formal analysis. **E.K. Hlil:** Writing – review & editing, Validation, Software, Investigation, Data curation.

## Declaration of competing interest

The authors declare that they have no known competing financial interests or personal relationships that could have appeared to influence the work reported in this paper.
